# Highly effective removal of perfluorooctanoic acid (PFOA) in water with DBD-plasma-enhanced rice husks

**DOI:** 10.1038/s41598-023-40197-3

**Published:** 2023-08-14

**Authors:** Thera Sahara, Doonyapong Wongsawaeng, Kanokwan Ngaosuwan, Worapon Kiatkittipong, Peter Hosemann, Suttichai Assabumrungrat

**Affiliations:** 1 Research Unit on Plasma Technology for High-Performance Materials Development, Department of Nuclear Engineering, Faculty of Engineering, Chulalongkorn University, 254 Phayathai Road, Pathumwan, 10330 Bangkok, Thailand; 2 Division of Chemical Engineering, Faculty of Engineering, Rajamangala University of Technology Krungthep, Bangkok, 10120 Thailand; 3 Department of Chemical Engineering, Faculty of Engineering and Industrial Technology, Silpakorn University, Nakhon Pathom, 73000 Thailand; 4 Department of Nuclear Engineering, Faculty of Engineering, University of California, Berkeley, 94720 USA; 5 Center of Excellence in Catalysis and Catalytic Reaction Engineering, Department of Chemical Engineering, Faculty of Engineering, Chulalongkorn University, Bangkok, 10330 Thailand; 6 Bio-Circular-Green-Economy Technology & Engineering Center (BCGeTEC), Faculty of Engineering, Chulalongkorn University, Bangkok, 10330 Thailand

**Keywords:** Environmental impact, Engineering

## Abstract

Adsorption is regarded as an efficient method to eliminate per- and polyfluoroalkyl substances from an aqueous solution. In the present investigation, an adsorbent based on rice husks (RHs) was successfully prepared by phosphoric acid (PA) activation and dielectric barrier discharge (DBD) plasma treatment, and it was used to adsorb perfluorooctanoic acid (PFOA) from water. The electrodes employed in the experiment were planar type. This research investigated RH surface properties and adsorption capacity before and after modification using DBD plasma. The results revealed that the He–O_2_ plasma modification introduced oxygen-containing functional groups and increased the PFOA removal efficiency. Increasing the oxygen content and total gas flow rate to 30 vol.% and 1.5 L/min, respectively, with 10 min of RH plasma treatment time at 100 W plasma discharge power enhanced the PFOA removal efficiency to 92.0%, while non-treated RH showed the removal efficiency of only 46.4%. The removal efficiency of the solution increased to 96.7% upon adjusting the pH to 4. The adsorption equilibrium isotherms fitted the Langmuir model, and the adsorption kinetic followed the pseudo-second-order model. The maximum adsorption capacity was 565 mg/g when the Langmuir isotherm model was applied.

## Introduction

Recently, freshwater resources faced significant contamination due to the release of dangerous substances such as per- and polyfluoroalkyl substances (PFAS)^1^ into the environment through man-made sources. PFAS are a group of chemicals containing hydrophobic chains fully or partially saturated with fluorine atoms. They are used in various consumer and industrial products such as pulp and paper, surface coating, textiles, and fire-fighting foams^2^. PFAS are known of being highly persistent (half-life > 92 years in water at 25 °C), potentially toxic, and bioaccumulative. The unique amphiphilic properties with thermal and chemical stability make PFAS harmful chemicals to the environment^3–5^.

PFAS can be differentiated based on their terminal functional groups, which are per-fluoroalkyl carboxylic acids (PFCA) and perfluoroalkyl sulfonic acids (PFSA). The most often detected PFCA and PFSA groups are perfluorooctanoic acid (PFOA) and perfluorooctanesulfonic acid (PFOS), respectively. The physicochemical properties of PFAS are directly associated with their chain length (C_n_F_2n_ _+_ _1_), with long-chain PFCA having 8 or more carbons and long-chain PFSA having 6 or more carbons^6^. PFCA with 7 or fewer carbons and PFSA with 5 or fewer carbons belong to short-chain PFAS^7,8^. The increasing carbon chain length of PFAS will increase bioaccumulation and biomagnification in the environment^9^.

A new and efficient method for removing organic pollutants in water reservoirs is urgently needed. Nonetheless, the removal of PFAS from already contaminated water reservoirs still lacks attention. Some methods have been investigated by researchers, for example, ion exchange, chemical coagulation, and adsorption techniques using activated carbons^10^. These methods, however, are not as effective as one would hope, especially for organic pollutants and the possibility to produce unexpected compounds during the removal process^11^. Therefore, developing a novel method for effectively removing PFAS in water is desired. The adsorption method is considered an effective technique compared to other physicochemical technologies in eliminating PFAS from water because it has a convenient operational design, low installation cost, and high performance^3,12^. Schroder et al.^13^ performed comparisons between chemical and physical methods for PFOA and PFOS removal. It was discovered that the adsorption method with granular activated carbon (GAC) is remarkably good compared to other conventional methods like the advanced oxidation process and reverse osmosis^13^.

The plasma technique presents an efficient method for the surface modification of materials^14^. As the process is convenient and environmentally friendly, some researchers have utilized plasma techniques to modify carbon-based materials^14^. Another important aspect of this method is its high efficiency and ability to evade secondary pollution^4^. Two main processes are involved in plasma surface modification: physical and chemical. The physical process can physically modify the surface structure of materials, meanwhile, the chemical process can present functional groups onto material surfaces^14,15^. The enhancement of the material’s porous structure can be influenced by physical effects such as shock waves, collision, and ultraviolet light irradiation which might be advantageous for the adsorption process^16^. Park et al.^17^ have revealed that chemical effects from atmospheric pressure plasma treatment can generate oxygenated functional groups on the surface of the activated carbon fibers. Consequently, modifying adsorbent materials using non-thermal (low-temperature) plasma is beneficial for improving adsorbability. It was found that the oxygen plasma treatment of activated carbon provides some potential benefits compared to conventional carbon activation methods as follows: (1) changes in surface qualities observed at low burnoff result in less material use and (2) altering the nature of the plasma gas carrier makes it feasible to adjust chemical characteristics of activated carbon^18^. While several plasma-treated carbon-based materials have been reported, the impact of dielectric barrier discharge (DBD) plasma has received little attention on adsorbent materials; therefore, this research focused on DBD plasma’s effects on the adsorption characteristics of the adsorbent.

Biosorption, which involves the use of biomass from agricultural materials, is a key area of focus within the adsorption method. It has the ability to remove organic contaminants in the environment by generating activated carbons through physicochemical and metabolically independent processes^19,20^. It should be noted that most agricultural wastes are usually utilized after being modified in various ways to improve the adsorption capacity, such as increasing the porosity and adsorption area, as well as retaining the suitable average pore size, instead of the original state^7^. Some agricultural wastes such as orange peels^21^, banana peels^22^, sawdust^23^, and rice husks^24^ have previously been investigated as biosorbents to remove pollutants.

The present investigation utilized agro-based rice husk (RH) as feedstock to produce plasma-modified biosorbents. The estimated rice production worldwide was approximately 517.1 million tons in 2021^25^, which gives a virtually unlimited zero-cost raw material resource for scientific studies^26^. Since RH is abundant, contains several functional groups, and is a no-cost agricultural by-product, much research effort has been on the preparation of effective biosorbents to remove organic contaminants. A study conducted by Daffalla et al.^27^ investigated various treatments on RHs and evaluated their effectiveness in removing phenol. According to their findings, when RHs were modified solely through thermal treatment, the phenol removal efficiency ranged from 36 to 64%. On the other hand, chemical treatment alone resulted in a lower removal efficiency of 28%.

In the present study, RH has been used to prepare the adsorbent for PFOA removal. RH was treated with He–O_2_ plasma generated in a planar-type DBD plasma reactor. The presence of He facilitated the generation of a spatially uniform plasma^28^. The He–O_2_-based DBD plasma offers outstanding advantages for the adsorbent treatment^28^. When oxygen is present in the gas mixture, the discharge generates oxygen radicals on the adsorbent, leading to the creation of acidic surface groups. These groups include carboxylic acid groups, derivatives of carboxylic acid groups such as carboxylic anhydride, lactone, and lactol groups, as well as phenolic groups that can be useful for adsorption^28,29^. Among several parameters investigated, the effect of different carrier gases was evaluated because dissimilar gases provide different characteristics of the modified material. Phosphoric acid was chosen as the activating agent before RH was plasma-treated because it is beneficial for the creation of the cross-linked structure with both micropores and mesopores^30^. The adsorption mechanism at different solution pHs, adsorption isotherms, adsorption kinetics, and possible interactions between RHs and PFOA have been studied and discussed. The objective of this study is to present a comprehensive study on the preparation, characterization, and adsorption performance of plasma-modified RHs as a potential high-impact solution for PFOA removal. To the best of our knowledge, DBD-plasma-enhanced RHs to remove PFOA from an aqueous solution were not studied previously and, therefore, present a novel approach with a potentially high impact.

## Materials and methods

### Materials

RHs from white rice were obtained from a local market in Bangkok, Thailand. Perfluorooctanoic acid (PFOA) was purchased from Sigma Aldrich with a molecular weight of 414.07 g/mol. 85% phosphoric acid (H_3_PO_4_, PA) was procured from Anapure. Hydrochloric acid (HCl) from QRëC and sodium hydroxide (NaOH) from Sigma Aldrich were used to control the pH solution. 99.9% sodium chloride (NaCl) was purchased from Gammaco for pH point of zero charge (pH_pzc_) determination. A glass fibers syringe filter with a 2.0 µm pore size was purchased from Merck Millipore. 0.1 N sodium thiosulfate (Na_2_S_2_O_3_) with a molecular weight of 248.18 g/mol was obtained from KemAus for the titration method. The corn starch for the starch solution was acquired from a nearby supermarket. Wijs iodine solution was procured from RCI Labscan. All required solutions were prepared using deionized (DI) water generated in the laboratory. Ultra-high purity (UHP) He and O_2_ gas cylinders were procured from Alternative Chemical.

### Activated RHs with phosphoric acid (PA)

The RHs were submerged in DI water for approximately 4 h and subsequently rinsed to eliminate any impurities and suspended particles. Afterward, they were dried in an oven at a temperature of 50 °C for 24 h before being immersed in 1 M PA solution for 24 h. The mixture was filtered and rinsed thoroughly with DI water and the residue was oven-dried at 105 °C until a consistent weight was obtained. Finally, the activated RHs were turned into a fine powder using a household blender and were sieved to gain particle sizes of 0.–0.3 mm. The same PA-activated RHs generated were used throughout the investigation.

### DBD plasma treatment of PA-activated RHs

The power supply of the DBD plasma system was adopted from a neon transformer (adjustable output voltage of 0–15 kV, output current of up to 30 mA, and constant frequency of 25 kHz). The output power can be regulated via a manual voltage regulator (variac) connected to the neon transformer, which was maintained at 100–110 W for every experiment. The parallel-plate electrodes were made of aluminum with a discharge electrode size of 9.5 cm × 13.5 cm and a ground electrode size of 11 cm × 14 cm. The discharge electrode was installed inside a glass reactor with a glass dielectric material of 10 cm × 14 cm in dimensions attached to it. The reaction chamber was an 800 mL borosilicate glass container and was placed on top of the ground electrode. The discharge gap, the air space between the bottom of the glass dielectric material and the base of the glass reactor, was fixed at 3 mm for every experiment. The PA-activated RHs were treated with DBD plasma. The illustration of the DBD plasma system is demonstrated in Fig. S1a and the plasma glow discharge is shown in Fig. S1b in the Supporting Information (SI). The He–O_2_ gas mixture was introduced into the DBD plasma chamber with several ratios (0, 10, 20, and 30 vol.% O_2_) and flow rates (0.5, 1, and 1.5 L/min). The oxygen concentration was studied up to 30 vol.%, beyond which a flame occurred inside the reaction chamber due to the flammable properties of oxygen gas. Both of the gases were individually regulated with distinctive mass flow controllers (MFCs). The plasma treatment duration was 5–25 min. The plasma-treated RH samples were stored in airtight glass containers before the adsorption process.

### Adsorption experiment

For each batch adsorption experiment, 20 mg of the PA-activated plasma-treated RHs were added to a glass beaker containing a 100 mL PFOA solution with a starting concentration of 70 mg/L. The adsorption investigation was carried out at 180 rpm for 44 h on a hot plate stirrer equipped with a magnetic stirrer bar with a controlled temperature of 25 °C^31^. To assess the impact of solution pH, either HCl or NaOH was introduced to the solution to maintain a pH level within the range of 4.0–10.0. Each experiment was conducted twice, and the mean value with an error bar was reported. It should be noted that the proper handling of PFOA-adsorbed material is essential to prevent any potential release or contamination. It is recommended to store the material in sealed containers in a secure, well-ventilated area that is separated from incompatible substances. This helps ensure the containment and safety of the adsorbed material. Additionally, the collected waste should be appropriately disposed of through processes such as incineration at authorized waste management facilities.

In the adsorption isotherm experiment, 20 mg of PA-activated plasma-treated RHs were added to a 100 mL PFOA solution with a starting PFOA concentration in the range of 10–500 mg/L. Both Langmuir and Freundlich models were employed to describe the adsorption isotherm. The classical Langmuir and Freundlich models (Eqs. (1) and (2), respectively) are described in the following formulas^32^:

1 $$ q_{e} = \frac{{q_{\max } C_{e} K_{l} }}{{1 + C_{e} K_{l} }}, $$

2 $$ q_{e} = K_{f} C_{e}^{\frac{1}{n}} , $$

where q_e_ (mg/g) indicates the adsorption capacity; q_max_ (mg/g) denotes the maximum adsorption capacity of solid-phase concentration which corresponds to a fully covered monolayer of the adsorption sites; C_e_ (mg/L) is the equilibrium concentration; K_l_ (L/mg) is the Langmuir’s isotherm constant; K_f_ is the Freundlich's isotherm constant, and n is the Freundlich’s exponent representing the adsorption intensity.

The adsorption kinetic experiment was conducted by adding 20 mg of PA-activated plasma-enhanced RHs into 100 mL of 50 mg/L PFOA solution. The residual concentration of PFOA was observed at different times during the adsorption experiment starting from 0 to 42 h. The adsorption rate of RHs was calculated using pseudo-first-order, pseudo-second-order, intraparticle diffusion, and Boyd kinetic models. The pseudo-first-order model is expressed by Eq. (3)^33^:

3 $$ \ln \left( {q_{e} - q_{t} } \right) = \ln q_{e} - k_{1} t, $$

where q_t_ denotes the adsorption capacity (mg/g) at time t; q_e_ denotes the adsorption capacity (mg/g) at equilibrium, and k_1_ (min^−1^) is the pseudo-first-order sorption rate constant. The pseudo-second-order model is represented by Eq. (4)^33^:

4 $$ \frac{t}{{q_{t} }} = \frac{1}{{q_{e}^{2} + k_{2} }} + \frac{1}{{q_{e} }}, $$

where k_2_ (g mg^−1^ min^−1^) is the pseudo-second-order sorption rate constant. The linear coefficient regression (R^2^) values are utilized to identify the most suitable kinetic model for predicting the adsorption process. The main difference in the applicability of the two models is related to the availability of active sites in the adsorbent. The intraparticle diffusion model is given by Eq. (5)^34^. This model assumes that external mass transfer is negligible.

5 $${q}_{t} ={k}_{d }{t}^{1/2} +\mathrm{ C},$$

where k_d_ (mg/g h^1/2^) is the intraparticle diffusion rate constant; t^1/2^ (h^1/2^) is the square root of time, and C (mg/g) is the boundary layer thickness coefficient. k_d_ was determined from the slope of the plot of q_t_ versus t^1/2^*.* If the regression passes through the origin, only the intraparticle diffusion governs the adsorption process (external mass transfer is inconsiderable). In contrast, if the plot does not pass through the origin, several mechanisms participate in the rate-limiting step. The Boyd kinetic model was evaluated with Eqs. (6) and (7)^35^ to further define the real rate-controlling step in the entire adsorption process^36^.

6 $$ F = \frac{{q_{t} }}{{q_{e} }} = \left( {1 - \frac{6}{{\pi^{2} }}} \right)\exp - B_{t} , $$

7 $$ B_{t} = - 0.4977 - \ln \left( {1 - F} \right). $$

The variable F denotes the fractional achievement of equilibrium at a given time t, while B_t_ represents the Boyd constant. If the graph of B_t_ versus time is linear and passes through the origin, the rate-limiting process in the adsorption mechanism is intraparticle diffusion.

### PFAS determination

Following the adsorption experiment, the mixture was separated by filtration through a 2.0 µm syringe filter. Controlled experiments showed that the amount of PFOA adsorbed on the syringe filter was insignificant because of the high concentrations. High-Performance Liquid Chromatography (HPLC, Alltech) with a conductivity detector was used to determine the PFOA concentration. HPLC was furnished with a TC- C18 column (4.6 × 250 mm) from Agilent Technologies (USA). The mobile phase consisted of a mixture of 65% methanol and 35% NaH_2_PO_4_, and the volume of the sample was 20 µL. The PFOA detection limit was 0.5 mg/L.

### Characterization of plasma-treated RHs

The calculation of the iodine number was conducted following the procedure outlined in the ASTM D4607-94^37^. First, a 1% starch solution was synthesized by adding 0.5 g starch into 50 mL boiling DI water with stirring until the starch was completely dissolved. 10 mL of 5 wt.% HCl solution was added to each flask containing 0.1, 0.3, and 0.5 g of RHs. Afterward, 100 mL of 0.1 N Wijs iodine solution was added to every mixture by stirring for 30 s. The produced solution was filtered and the filtrate of 50 mL was titrated with 0.1 N Na_2_S_2_O_3_ until the solution became pale yellow^38^. Then, 2 mL of the starch indicator was introduced into the solution and the titration was continued with 0.1 N Na_2_S_2_O_3_ until one additional drop produced a colorless solution. The volume of 0.1 N Na_2_S_2_O_3_ that has been used was recorded.

The iodine number calculation was performed to determine the adsorbent’s uptake capacity, which is defined as mg of iodine adsorbed by 1 g of the adsorbent^39^. The iodine number determination is a precise way to estimate both the microporosity and surface area of an adsorbent^39^. The iodine number was calculated based on Eqs. (8) and (9) as follows^40^:

8 $$ \frac{X}{M} = \frac{{\left\{ {\left( {N_{1} \times 126.93 \times V_{1} } \right) - \left[ {\left( {V_{1} + V_{HCL} } \right)/V_{F} } \right] \times \left( {N_{Sodium\,\,\,Thiosulfate} \times 126.93} \right) \times V_{{_{Sodium\,\,\,Thiosulfate} }} } \right\}}}{{M_{c} }}, $$

9 $$ C = \left( {N_{Sodium\,\,Thiosulfate} \times V_{{_{Sodium\,\,Thiosulfate} }} } \right), $$

where *(X/M)* is the quantity of iodine adsorbed per gram of RHs; *C* is the iodine concentration in the filtrate; N_1_ represents the normality of the iodine solution; V_1_ represents the volume of the added iodine solution; V_HCl_ is the added volume of 5% HCl; V_F_ is the volume of the filtrate used during titration; N_Sodium Thiosulfate_ is the normality of sodium thiosulfate solution; V_Sodium Thiosulfate_ represents the volume of sodium thiosulfate solution that has been consumed, and M_C_ is the weight of RHs. The iodine number is expressed as the *X/M* value at a 0.02 N residual iodine concentration (*C*).

The pH drift method was employed to calculate the pH point of zero charge (pH_pzc_) of plasma-treated RHs. First, 0.01 M NaCl was prepared, and 50 mL of this solution was taken to a flask. The NaCl solution was boiled to get rid of the dissolved CO_2_ until a stable pH was obtained, and it was kept in an air-tight condition. If the solution is exposed to air, the dissolution of CO_2_ can lead to a reduced pH. The solution pH was tailored by adding a few drops of HCl or NaOH to obtain an initial pH in the range of 2–8. 150 mg of plasma-treated RHs was added into the flask containing 50 mL of the NaCl solution with 1500 rpm of stirring for 15 h at ambient temperature. Afterward, the solution was filtered with the final pH for each set measured.

Fourier-transform infrared (FT-IR) spectroscopy was utilized to investigate the chemical bonds of the RH samples. The surface morphology alteration of RHs from the plasma application was observed with a scanning electron microscope and energy-dispersive X-ray spectrometer (SEM–EDS; JEOL JSM-IT300). X-ray photoelectron spectroscopy (XPS; AXIS Ultra DLD) analysis was conducted to examine the detailed surface chemical composition between untreated and plasma-treated RHs at different feeding gas compositions.

The texture of the PA-activated DBD plasma-treated RH samples under different plasma treatments was characterized by analyzing the N_2_ adsorption isotherms acquired at 77 K using a BET analyzer (ASAP 2460, Micromeritics, USA) to identify the physical structures. To determine the total pore volume, the quantity of N_2_ adsorbed at a relative pressure of P/P_o_ ~ 0.99 was employed. The total pore volume is the quantitative measure of the volume of all porous spaces within a particle, including all internal void spaces, regardless of pore width^41^. The calculation of the average pore diameter (D) was performed by presuming a cylindrical shape of pores from the BET surface area (S_BET_) and total pore volume (VT), D = 4VT/S_BET_^42^.

## Results and discussion

### FTIR analysis

**Figure 1:**
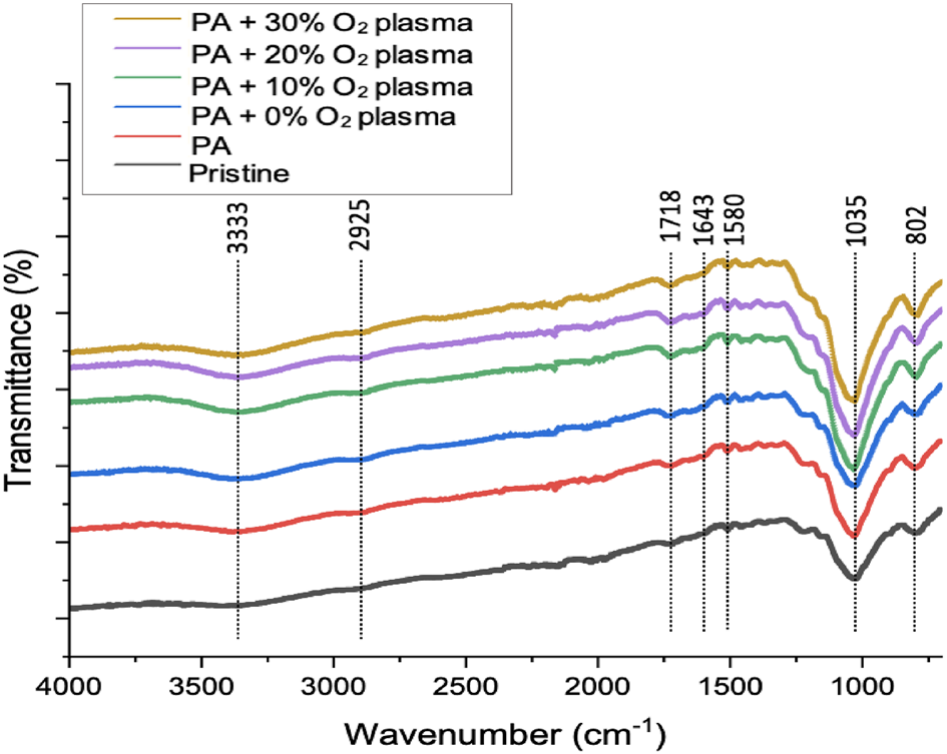
FTIR spectra of untreated RHs (Pristine), PA-activated RHs (PA), and PA-activated RH + He-O_2_ plasma treatment with a% oxygen (PA + a% O_2_ plasma) under 10 min plasma application time at 100 W and 1.5 L/min total gas flow rate (a% denoting O_2_ percentage in He-O_2_ gas mixture).

Figure 1 depicts the FTIR spectra of RHs under various treatment conditions: untreated RHs (Pristine), PA-activated RHs (PA), and PA-activated RHs + He-O_2_ plasma treatment with various O_2_ concentrations from 0 to 30 vol.% (PA + a% O_2_ plasma) under 10 min of plasma application time and 100 W plasma discharge power. The total gas flow rate was set at 1.5 L/min for every plasma treatment condition. RHs exhibited a peak at 3333 cm^–1^ indicating O–H stretching or the hydroxyl group of cellulose and hemicellulose in plant fibers^43^, a peak at 2925 cm^–1^ attributing to C–H stretching or alkane^44^, a peak at 1718 cm^–1^ indicating C=O (–COOH) or carboxylic acid^45^, a peak at 1643 cm^–1^ representing C=C or alkene^46^, the adsorption band at 1580 cm^–1^ due to C–C stretching vibration in the aromatic ring^47^, a strong absorption peak at 1035 cm^–1^ representing C–O stretching of cellulose^48^, and a peak around 802 cm^–1^ indicating symmetric vibration of the silanol bond (Si–O)^49^.

These results demonstrate that the functional groups containing oxygen present on the RH surfaces were dependent on the studied treatment method. Pristine RHs show the lowest intensity of oxygenated functional groups compared to the treated RHs, where the highest intensity of the functional groups was obtained for the PA + 30% O_2_ plasma sample as depicted in Fig. 1. Thus, the DBD plasma treatment with higher oxygen concentration can create more abundantly the oxygenated functional groups associated with the molecular bond. Due to the penning reactions (Eqs. 10 and 11) taking place in the discharge region, it can help to generate atomic oxygen radicals by reducing the concentration of helium metastable species and then increasing the oxygen content^50^. In plasma, the contribution of electron-O_2_ collisions to O_2_ dissociation is significant and plays a crucial role in various plasma processes. Plasma is a highly ionized and energetic state of matter consisting of free electrons, ions, excited and neutral species. When highly energetic electrons collide with O_2_ molecules, they can transfer sufficient energy (the transferred energy exceeds the dissociation energy of the O_2_ bond) to break the O_2_ bond and dissociate the molecule into two oxygen atoms (O)^51,52^. This dissociation process is known as electron impact dissociation^51^.

Throughout the discharge of the He–O_2_ plasma, oxidation may occur at either the aromatic ring or the substituents of RHs. Then, the oxygen radicals generated during the discharge assault the aromatic system by adding to the aromatic double bonds (C=C) ^28^. Meanwhile, from Fig. S2, it is apparent that variations of plasma treatment time from 5 to 25 min did not contribute to significant changes in the intensity and peak position. This indicates that prolonged treatment time did not change the chemical composition significantly, but it had more effect on physical conditions.

10 $$ {\text{He}}_{{\text{m}}} + {\text{O}}_{2} \to {\text{O}}_{2}^{ + } + {\text{He}} + {\text{e}}^{ - } , $$

11 $$ {\text{O}}_{2}^{ + } + {\text{e}}^{ - } \to 2{\text{O}}. $$

### XPS analysis

Figure S3 depicts the high-resolution XPS spectra with a specific range of binding energy of C1s. The C1s spectra can be separated into 4 characteristic peaks for each sample. The peaks at approximately 284.6, 286.1, 287.9, and 289.0 eV correspond to carbon bonds of –C–C– groups; ether, phenol, or alcoholic groups (C–O); carbonyl groups (C=O); and carboxyl or ester groups (O–C=O), respectively^53,54^. Table 1 summarizes the electron binding energies and intensities (derived by dividing the area of the small peak by the total area) of every peak, considering the typical uncertainty of quantitative XPS measurement of 10%^47^. As shown in Fig. S3, C–C and C–O are the main components of pristine RHs, while the carbonyl and carboxyl groups of pristine RHs account for only 5 and 6% respectively. The XPS results suggested that, as the oxygen gas concentration increased, the amount of C=O and COOH continued to increase by the plasma treatment, which was caused by high-energy electrons, highly active species, and thermal effect encouraging the conversion of carbonyl groups into carboxyl groups^47^. The highest contents of carbonyl and carboxyl groups in RHs were obtained at 18 and 19.6%, respectively, when the plasma feeding gas consisted of 30% O_2_ and 70% He, confirming that the plasma modification process successfully loaded elemental oxygen on the RH surfaces. The basal structure of the adsorbent surface can behave as soft centers that capture soft ions, while the oxygen-containing functional groups act as hard sites that can retain the hard ions and molecules^53^. Consequently, the RH adsorption capability improved as its oxygen concentration increased, which were validated by the adsorption assessment tests of PFOA onto RHs.

**Table 1:** The relative content of RH functional groups of pristine, PA-activated, and PA-activated plasma-modified RHs from XPS spectra of C1s (10 min plasma treatment time, 100 W, and 1.5 L/min total gas flow rate).

Functional group	Binding energy (eV)	Relative intensity (%)					
		Pristine	PA	PA + 10% O_2_	PA + 20% O_2_	PA + 30% O_2_	PA + 100% He
C − C	284.6	50.7	34.4	33.9	23.4	17.3	34.9
C − O	286.1	38.3	46.7	45.4	48.6	45.1	44.0
C = O	287.9	5.0	9.5	12.5	13.2	18.0	13.6
COOH	289.0	6.0	7.4	8.2	14.8	19.6	7.5

### Brunauer–Emmett–Teller (BET) analysis

The BET surface areas, average pore diameter, and total pore volume of PA-activated plasma-treated RHs were presented in Table 2. Surface area is one of the important adsorption properties because it is directly proportional to adsorption capacity^55^. The findings reveal that the BET-specific surface area and total pore volume of untreated RHs were the lowest, while the values for plasma-treated RHs gradually increased during the first 10 min of plasma exposure which could improve the accessibility of PFOA molecules to the pores. However, when the exposure time was prolonged from 10 to 25 min with the same oxygen content and total gas flow rate (30 vol.% and 1.5 L/min, respectively), the surface area was reduced from 1196 to 1033 m^2^/g because of the stronger etching effect leading to the destruction of pore walls. The reduction of the surface area can also be caused by the blockage of pores due to surface functional groups; nonetheless, the existence of surface functional groups can improve the adsorbability by providing more hydrogen ions^28^. Other RH modification methods have been studied such as chemical treatment by calcium hydroxide, thermal treatment, and CO_2_ activation with a surface area of 3.19, 201, and 502 m^2^/g, respectively^56,57^. Compared to these results, the plasma-treated RHs achieved the largest surface area. The complex porous structure of RHs indicates that the specific surface area is fundamentally governed by the internal porous structure^53^. Consequently, the alteration of the total pore volume directly affected the surface area, as when the total pore volume increased to 0.997 cm^3^/g, the specific surface area reached the maximum value.

**Table 2:** Structural properties of RHs under various plasma modification times (PA + 30% O_2_ plasma, 100 W, and 1.5 L/min total gas flow rate).

Sample	S_BET_ (m^2^/g)	Total pore volume (cm^3^/g)	Average pore diameter (nm)
Untreated	858	0.74	3.44
5 min	1023	0.89	3.49
10 min	1196	0.99	3.34
15 min	1129	0.93	3.28
20 min	1094	0.91	3.31
25 min	1033	0.88	3.39

When RH was exposed to plasma, several processes occurred simultaneously that contributed to the modification of its surface and internal structure. These processes included physical effects such as shock waves, collision, and ultraviolet light irradiation, as well as chemical effects resulting from reactive species generated in the plasma. These effects contributed to the enhancement of surface properties. Shock waves create mechanical stress, resulting in surface cracking, fragmentation, and the formation of new surface features, thereby increasing the surface area^58^. Collision of energetic particles with the RH surface causes physical sputtering, exposing surfaces and contributing to an increased surface area^59^. The plasma’s UV light irradiation initiates photochemical reactions, breaking chemical bonds and leading to the formation of new surface structures and functional groups^60^.

Chemically, the reactive species present in the plasma, such as ions, radicals, and excited molecules, played a crucial role. They chemically reacted with the RH surface, forming new chemical bonds and functional groups. Oxygen radicals, specifically, reacted with the RH surface, introducing oxygen-containing functional groups like carboxylic acids, which enhanced adsorption capacity and reactivity. Moreover, the plasma treatment induced cross-linking reactions that involved the reaction of reactive species present in the plasma with the surface of the RH. These reactive species, such as ions, radicals, and excited molecules, can chemically react with the surface, causing the bonds formation between molecules^59^. As the cross-linking reactions progressed, the interconnected network of molecules bonds expanded, leading to the creation of larger pores volume^61^.

### SEM analysis

**Figure 2:**
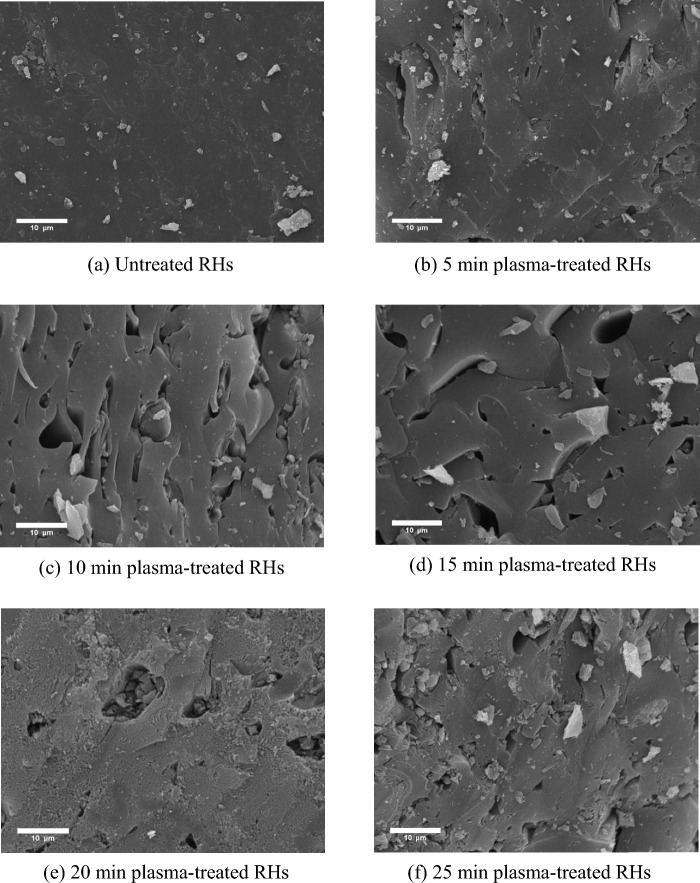
SEM micrographs of RHs with various plasma treatment times for PA + 30% O_2_ plasma at 100 W and 1.5 L/min total gas flow rate (5000 × magnification).

The influence of He-O_2_ plasma on the surface characteristics of RHs was examined at different plasma exposure times from 0 to 25 min for PA + 30% O_2_ plasma at 100 W of plasma discharge power and 1.5 L/min total gas flow rate. The SEM micrograph of untreated RHs indicated a relatively smooth surface (Fig. 2a). The treated RHs showed more cracks, small cavities, and open pores (Fig. 2b–f), supporting the earlier finding that the treated RHs exhibited higher surface area than untreated ones^62^. Raising the plasma treatment time contributed to the surface roughness. Figure 2b illustrates that the surface of 5 min plasma-treated RHs started to become rough and uneven while the structure was still less porous compared to the longer plasma exposure time. Prolonging the plasma modification time to 10 min (Fig. 2c) enlarged the pore size with many craters and cavities of various sizes, along with increased surface area. This was caused by remarkably energetic electrons and active free radicals attacking the external surface of RHs, thus, increasing the number of adsorption sites^63^. The porous structure is a significant factor for physical adsorption, for PFOA with large molecular weights could get into the developed inner pores of the RHs. Nevertheless, when the RHs were plasma-treated for more than 15 min, the plasma destroyed the micropores, created pore blockage, and formed numerous fine particles (Fig. 2e). More cracks were created when the RHs were modified for 25 min (Fig. 2f) as the surface area was reduced. These results clearly demonstrated that the plasma modification has the potential impact on the external surface structure of RHs on the micron scale as corresponding to the BET analysis results^14^. Extending the plasma modification time reinforced the etching to become more violent and increased the functional group grafting effects^64^. Both of these effects work independently and they are opposite to each other, but plasma treatment mainly performs based on the association of these two effects^64^.

### Effects of total gas flow rate and O_2_ concentration on removal efficiency

**Figure 3:**
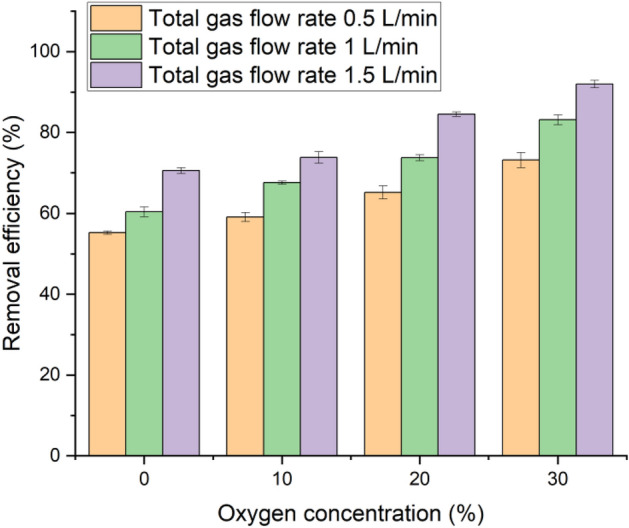
Effects of total gas flow rate and oxygen concentration on PFOA removal efficiency (10 min treatment time at 100 W).

As the primary objective of the present investigation is on PFOA removal from water, the effects of total gas flow rate and O_2_ concentration on the removal efficiency must be evaluated. The He-O_2_ feed gas mixture with different O_2_ concentrations and total gas flow rates was introduced into the reactor at atmospheric pressure to treat PA-activated RHs for 10 min before conventional batch adsorption was conducted. Figure 3 reveals the influence of the total gas flow rate and O_2_ concentration on PFOA removal efficiency. The maximum studied flow rate of 1.5 L/min resulted in the highest PFOA removal efficiency from the solution while increasing oxygen gas concentration in the plasma reactor also provided the same effect. The gradual increase of the total gas flow rate to 1.5 L/min introduced more gas molecules to be ionized, so the number of charged particles became higher. However, in the present experimental setup, the maximum total gas flow rate was observed at 1.5 L/min because a higher flow rate resulted in an unstable plasma. The lowest PFOA removal efficiency of 55.23% was observed when the reactor was fed only by helium gas at the total gas flow rate of 0.5 L/min. When 30 vol.% oxygen was introduced into the reactor with a total flow rate of 1.5 L/min, the highest removal efficiency of 91.96% was achieved. The presence of a small amount of oxygen in a helium DBD plasma reactor can result in a more uniform treatment to the RHs surface, as compared to pure oxygen plasma, random streamers are frequently observed in the discharge region and necessitate a higher discharge voltage^28^. Adding oxygen to the gas mixture allows the discharge to produce oxygen radicals, which react with the surface of RHs, leading to the formation of acidic functional groups that can effectively adsorb PFOA^28^. Figure S4 proposes the two-step oxidation of hydroxyl (–OH) groups of the glucose monomers of RHs cellulose. The first oxidation is the formation of aldehyde (–CHO), and then the aldehyde is oxidized further to form carboxylic acids (–COOH)^65^. It can be concluded that the amount of O-containing groups increased as the total gas flow rate increased, as well as increasing oxygen concentration, during the He–O_2_ plasma modification process, which is favorable for PFOA adsorption as the highest removal efficiency was achieved.

The corresponding temperatures when the change in the gas flow rate was made are presented in Fig. S5. As the gas flow rate increased, the gas temperature also increased. This can be attributed to the fact that a higher gas flow rate resulted in increased kinetic energy and collision frequency among gas molecules within the plasma system^66^. When the gas molecules collided, energy was transferred between them, leading to an overall increase in temperature^66^. This increased temperature can enhance the reactivity and effectiveness of the plasma treatment process.

The higher gas flow rate facilitated the transport of reactive species and energetic particles generated within the plasma to the surface of the RH. This increased transport rate ensured more efficient and rapid interaction between the plasma species and the RH. Consequently, the plasma treatment with a high gas flow rate can lead to improved removal efficiency of RH, as the reactive species had a higher chance of reaching and reacting with the RH surface.

In this study, the role of hydrogen bond (H–bond) interaction significantly influences the PFOA adsorption process through the interaction between oxygenated functional groups on the RH surface and the carboxylates head of PFOA by the creation of negative charge-assisted hydrogen bond (–) CAHB^67^. Normally, the hydrogen bond of solute-water and water-surface exists in intense competition with solute-surface H-bonds when water is abundant^68^. However, the existence of (–) CAHB can contribute to creating a strong hydrogen bond between the oxygenated functional groups and carboxylic acid of PFOA rather than the RH-water H-bond^68,69^. This interaction can be written as follows:

12 $$ {\text{RCOO}}^{ - } + {\text{H}}^{ + } +^{ - } {\text{O}} - {\text{RH}} \to \left( {{\text{RCOO}} \ldots {\text{H}} \ldots {\text{O}} - {\text{RH}}} \right)^{ - } . $$

In this respect, the pK_a_ equalization between proton donor DH (O functional groups) and proton acceptor AH (adsorbate) is responsible for the formation of (–) CAHB^69^. pKa is a numerical value that represents the degree of ease with which an acid donates its proton (H +) in a solution, and is therefore a measure of the acid's strength. The strength of the (–) CAHB becomes stronger as $\Delta$ pK_a_ value decreases, where $\Delta$ pK_a_ = pK_a(DH)_–pK_a(AH+)_ is defined as the discrepancy in acidic constants of the donor and acceptor heteroatoms (O or N), and the strength reaches a maximum when ΔpK_a_ nears zero or small negative values, depends on the surrounding conditions^69^. As RHs were treated with phosphoric acid (PA) and as the acid protonated the functional groups of the RHs, the pK_a_ of the RHs should be approximately the same as that of PA at around 2.1^70^. pK_a_ of PFOA was 2.5^71^ as shown in Table S1, so ΔpK_a_ in this experiment was − 0.4 which was very close to zero. This value supports the formation of (–) CAHB to improve the hydrogen bonding between RHs and oxygen atoms present in the functional head of PFOA that can act as acceptors.

### Effect of plasma modification duration on removal efficiency

**Figure 4:**
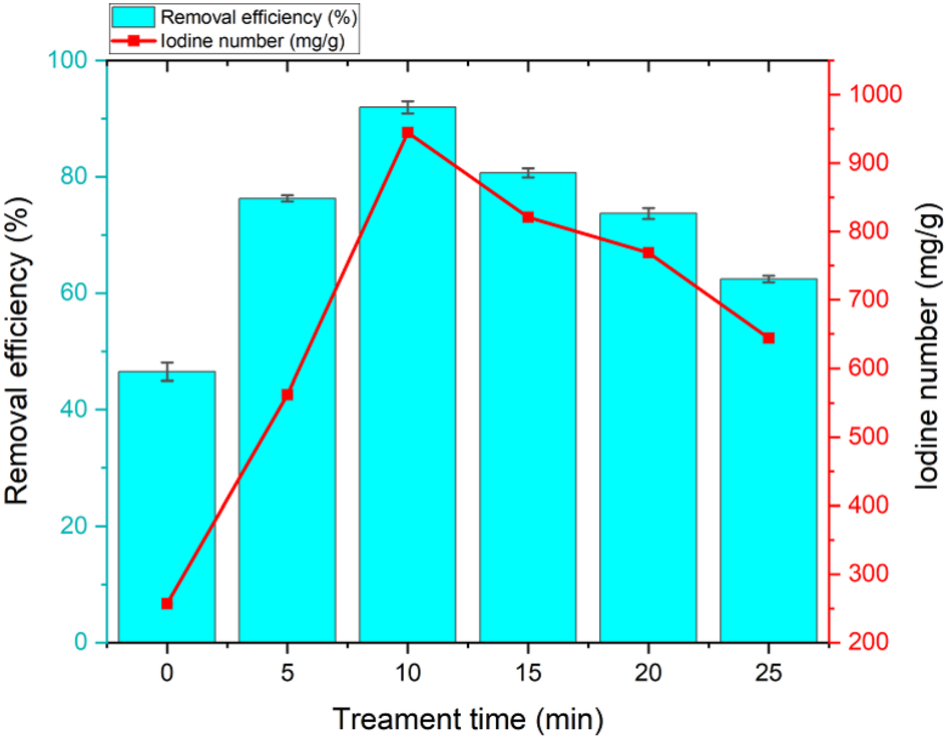
Effect of plasma treatment time on PFOA removal efficiency and iodine number (PA + 30% O_2_ plasma, 1.5 L/min total flow rate, 100 W, solution pH = 6).

Figure 4 illustrates the effect of plasma modification time on the removal efficiency of PFOA and iodine number. The modification time of 0 min represents PA-treated RHs without plasma treatment.

The experimental results demonstrate that the residual PFOA concentration after the adsorption process using PA-activated plasma-modified RHs was significantly lower than that of the PA-activated RHs (at 0 min). The removal efficiency of the PA-activated RHs without plasma exposure was only 46.4%. The PA-activated RHs modified for only 5 min triggered a sharp increase in the adsorption capacity. With increasing plasma exposure time from 5 to 10 min, the removal efficiency of PFOA in the solution drastically rose from 76.28 to 91.96%. The results indicate that 10 min is the optimal plasma treatment time and that a further increase to 25 min had a deteriorating effect on the uptake capacity as the removal efficiency decreased to 62.39%. The BET surface area and SEM micrograph results of the plasma-treated PA-activated RHs samples also correspond to the adsorption efficiency, indicating that the appropriate use of DBD plasma to modify PA-activated RHs can effectively improve its PFOA adsorption capacity. Excess plasma treatment duration may have caused the plasma to break the structure of RHs, reducing the surface area for adsorption^63^. These results are in accordance with the iodine number values, where the highest iodine number was 945 mg/g obtained from the PA-activated RHs treated with plasma for 10 min. PA-activated RHs without plasma treatment showed the lowest iodine number of 257 mg/g, confirming that non-plasma-treated RHs exhibited the lowest surface area for adsorption.

The critical micelle concentration (CMC) of PFOA was reported at around 15,696 mg/L^72^ as shown in Table S1. Although the CMC value of PFOA is high, it remains feasible for hemi-micelles and micelles to form on adsorbent surfaces when their concentrations are within 0.001–0.01 times of CMC^73^. The CMC is interpreted as the concentration of surfactants in a solution at which micelle formation is first observed^74^. PFOA tends to create hemi-micelles and micelles in water through the hydrophobic aggregation of C–F chains (Fig. S6)^72^. The morphology of PFOA hemi-micelles/micelles can be influenced by the surface characteristics of adsorbents via electrostatic attraction^67^.

In addition, the generation of micelles and hemi-micelles could enhance the adsorption into plasma-treated RH pores because of the accumulation of PFOA on the surface of RHs. The large pores of RHs facilitate more rapid external diffusion of PFOA; therefore, the mesopores of the adsorbent are better than micropores for PFOA diffusion because this formation has the potential to obstruct PFOA diffusion pathways into the inner surfaces of the RHs^36,75^. In this case, the 10 min plasma-treated PA-activated RHs are most favorable for the penetration of hemi-micelles/micelles into the pores because of the highest iodine number (largest surface area). When the plasma treatment became too long, the pore walls were destroyed by the plasma resulting in micelles and hemi-micelles being unable to penetrate the RH pores.

### Effect of solution pH on removal efficiency

**Figure 5:**
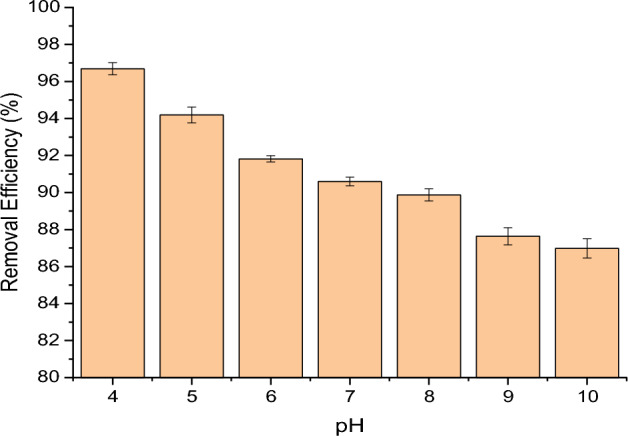
Effects of solution pH on PFOA removal efficiency (He + 30 vol.% O_2_, 1.5 L/min total flow rate, 10 min, and 100 W).

As mentioned before, the adsorption of PFOA on RHs is electrostatic in nature. Therefore, the influence of the solution pH on the adsorption of PFOA was examined under the optimal condition determined earlier (He + 30 vol.% O_2_, 1.5 L/min total flow rate, 10 min plasma treatment time, and 100 W plasma power) with the results illustrated in Fig. 5. Optimizing the adsorption of PFOA heavily relies on the solution pH, which is one of the most critical parameters. When the solution pH was changed from 4 to 10, the PFOA removal efficiency was reduced significantly from 96.69 to 86.98%. These results were directly affected by the electrostatic interaction between anionic PFOA and the charged oxygen functional groups of the RHs. The existence of electrostatic interaction in the adsorption process has been confirmed in this pH parameter investigation. The protonated surface of the adsorbent supports the electrostatic interaction by making compounds with negative charges, thus, anionic PFOA can be adsorbed readily. The negative charge of PFOA is not restricted to its functional groups. Due to the high electronegativity of fluorine atoms, PFOA molecules possess a positively charged core surrounded by a negatively charged shell; hence, it has the ability to attract electrons to an atom^76^. The electrostatic negativity of PFOA mainly arises from its functional head, whereas the tail of PFOA molecules primarily demonstrates the hydrophobic effect, which overwhelms the charge effect^67^.

pH_pzc_ has a substantial role in the electrostatic adsorption phenomenon as it describes a condition when the net charge density on the surface of RHs is zero/neutral (containing as many positively charged as negatively charged surface functions). The pH_pzc_ of the RHs obtained from this experiment can be derived from Fig. S7a by the intersection between the plot of the experimental data and the linear line, which is approximately 4.4. Since pKa of PFOA is 2.5^71^, it existed as anionic species at certain pHs^67^.

If the pH of the solution is below the pH_pzc_ of the adsorbent, the adsorbent becomes positively charged and develops more basic sites, or it can be concluded that the RH surfaces have protonated functional groups favoring PFOA adsorption due to electrostatic attraction. This protonated event can compress the electrical double layers of the adsorbent, weakening the electrostatic repulsion between RH surfaces and PFOA, as well as the electrostatic repulsion between PFOA anions^77^. The positively charged functional groups present on the RH surfaces exert an electrostatic attraction toward the anionic functional heads of PFOA molecules (Fig. S7b). In contrast, when the solution pH is higher than pH_pzc_ of the adsorbent, the adsorbent is deprotonated (losing protons) and becomes negatively charged (Fig. S7c). Then, the electrostatic attraction did not occur (electrostatic repulsion becoming stronger) resulting in decreased PFOA adsorption. The illustration of protonated and deprotonated mechanism is shown in Fig. S8. However, the adsorbed amount was still high indicating that other interactions were still involved^67^. Taking into account the hydrophobicity of PFOA, PFOA can be adsorbed on the RHs through hydrophobic interaction^78^. The hydrophobic interaction describes the affinity of nonpolar groups that have the propensity to create agglomeration in an aqueous solution^79^. It has been reported that the hydrophobic C–F chains of PFOA are also likely to disperse onto the negatively charged surface of an adsorbent at high pH^67^. The hydrophobic tails of PFOA are willing to aggregate on the surface of deprotonated RHs to form micelles or hemimicelles, whereas the polar head groups of PFOA are positioned at the interface between micelle and water^3^. However, the PFOA removal efficiency of deprotonated RHs is still lower than that of protonated ones because the electrostatic repulsion gives contribution to the adsorption. Based on the aforementioned observations, the adsorption mechanism of PFOA is contingent on the pH level. The hydrophilic region from the functional group head tends to create electrostatic interaction at pH < pH_pzc_ and a hydrophobic region from the perfluorinated tail tends to form hydrophobic interaction and hydrogen bond at pH > pH_pzc_. This finding solves the ambiguity of the PFOA adsorption mechanism because of its amphiphilic properties. If the solution pH = pH_pzc_, 50% of the functional groups will be protonated and 50% will be deprotonated. So the pH_pzc_ result is in accordance with this pH parameter study.

It is worth mentioning that plasma treatment can achieve these high removal efficiencies within a relatively short treatment time. By optimizing the process conditions and using a short treatment duration of only 10 min, it is possible to minimize the overall energy consumption and oxygen usage while still achieving the desired removal efficiency. This approach allows for the efficient use of resources and has the potential to reduce costs compared to longer treatment times.

The specific electrical energy consumed (S_EEC_) in unit removal of PFOA (kWh/mg) using PA-activated plasma-treated RHs as adsorbent was calculated using the following formula^80^:

13 $$ S_{EEC} = \frac{Pt}{{3.6 \times 10^{6} \times R \times C_{0} \times V}}, $$

where P is power consumption (W); t is time of operation (h); R is the removal efficiency; C_0_ is the initial concentration of PFOA (mg/L), and V is volume (L). The S_EEC_ obtained in this study was 0.68 kWh/kg, achieved under the optimal parameters of 100 W power consumption, 10 min plasma treatment time, a removal efficiency of 96.7%, an initial PFOA concentration of 70 mg/L, and a volume of 0.1 L. In comparison, Beiramzadeh et al.^80^ reported higher S_EEC_ values for heavy metals removal using the electrocoagulation method. The values of S_EEC_ of Fe and Ni were reported as 10.2 and 53.93 kWh/kg, respectively^80^.

### Adsorption isotherm study

**Figure 6:**
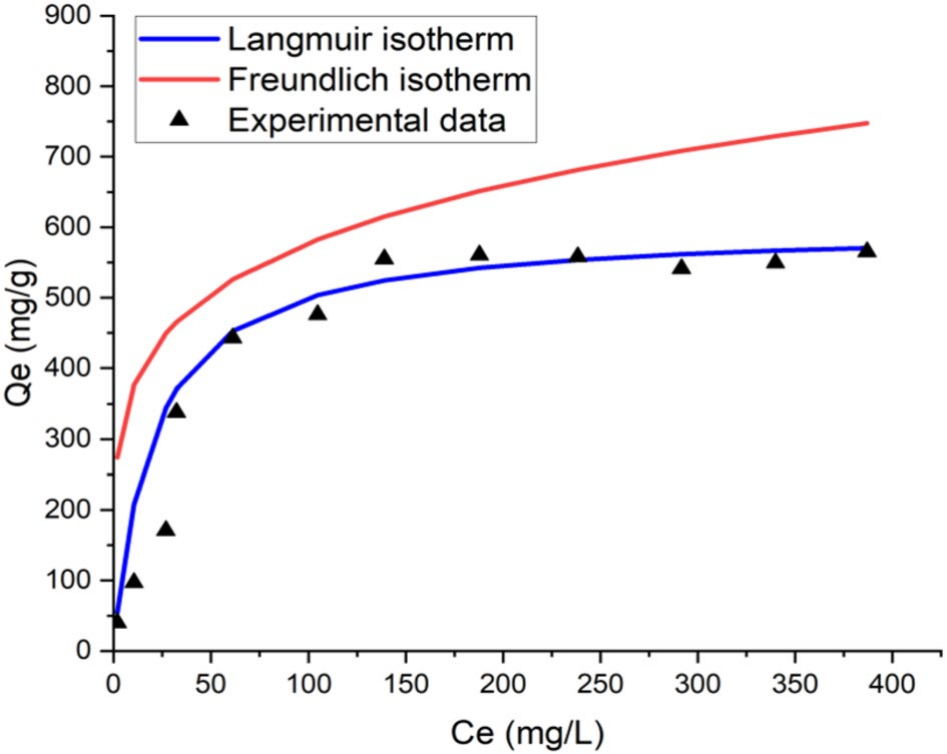
PFOA adsorption isotherms of RHs (initial PFOA concentration = 10–500 mg/L, adsorption time = 46 h, He + 30 vol.% O_2_, 1.5 L/min total flow rate, 10 min, and 100 W).

An adsorption isotherm is crucial to evaluating the uptake capacity of adsorbents and understanding the sorbate–sorbent interactions^72^. The adsorption capacity is not only influenced by the adsorbent properties but also by the solution pH^67^. Figure 6 presents the PFOA adsorption isotherms of the PA-activated RHs prepared from the optimal condition determined earlier (He + 30 vol.% O_2_, 1.5 L/min total flow rate, 10 min treatment time, and 100 W power).

Based on the Langmuir isotherm model, it shows that the PFOA molecules do not penetrate to other regions or layers of the RHs, then the adsorption process remains confined to a specific homogenous site^78^. The Freundlich adsorption isotherm assists to describe the multilayer adsorption process of PFOA onto RHs^78^.

The present study discovered that the Langmuir model described the PFOA adsorption isotherm better than the Freundlich model (Fig. 6). This aligns with the concept that PFOA adsorption by RHs adheres to the uniform energy and monolayer adsorption mechanism^81^. The parameters of the adsorption isotherm are presented in Table S2. The separation factor (R_L_) was less than the unity (0.261); therefore, the Langmuir model fitted the data adequately^82^. The maximum adsorption capacity was 565 mg/g with a correlation coefficient (R^2^) of 0.972 with the Langmuir isotherm model. The very high sorption may be associated with the highly hydrophobic C–F chains to form micelles and hemi-micelles^83^.

A literature review found that certain adsorbents have been employed to remove PFOA. The adsorption capacities of granular activated carbon (pH = 7.2), hexagonal mesoporous silica (pH = 6.8–7), zeolites (pH = 7), and *Vitis vinifera* leaf litter activated carbon were 112, 72.1, 88.8, and 78.9 mg/g, respectively^1,84,85^. The DBD plasma-treated PA-activated RHs show a much higher maximum PFOA adsorption capacity than the compared conventional adsorbents because the plasma treatment is highly effective in altering surface functionalities and morphology. The plasma treatment reactor is very simple and low cost, while the relevant process parameters such as plasma power, plasma treatment time, and feeding gas composition can be easily adjusted to obtain desired results.

### Adsorption kinetic study

The study on adsorption kinetic was investigated to obtain the resulting kinetic parameters and to gain some insight into the adsorption mechanisms. The PA-activated DBD plasma-treated RHs were prepared from the optimal condition determined earlier (He + 30 vol.% O_2_, 1.5 L/min total flow rate, 10 min treatment time, and 100 W power).

**Figure 7:**
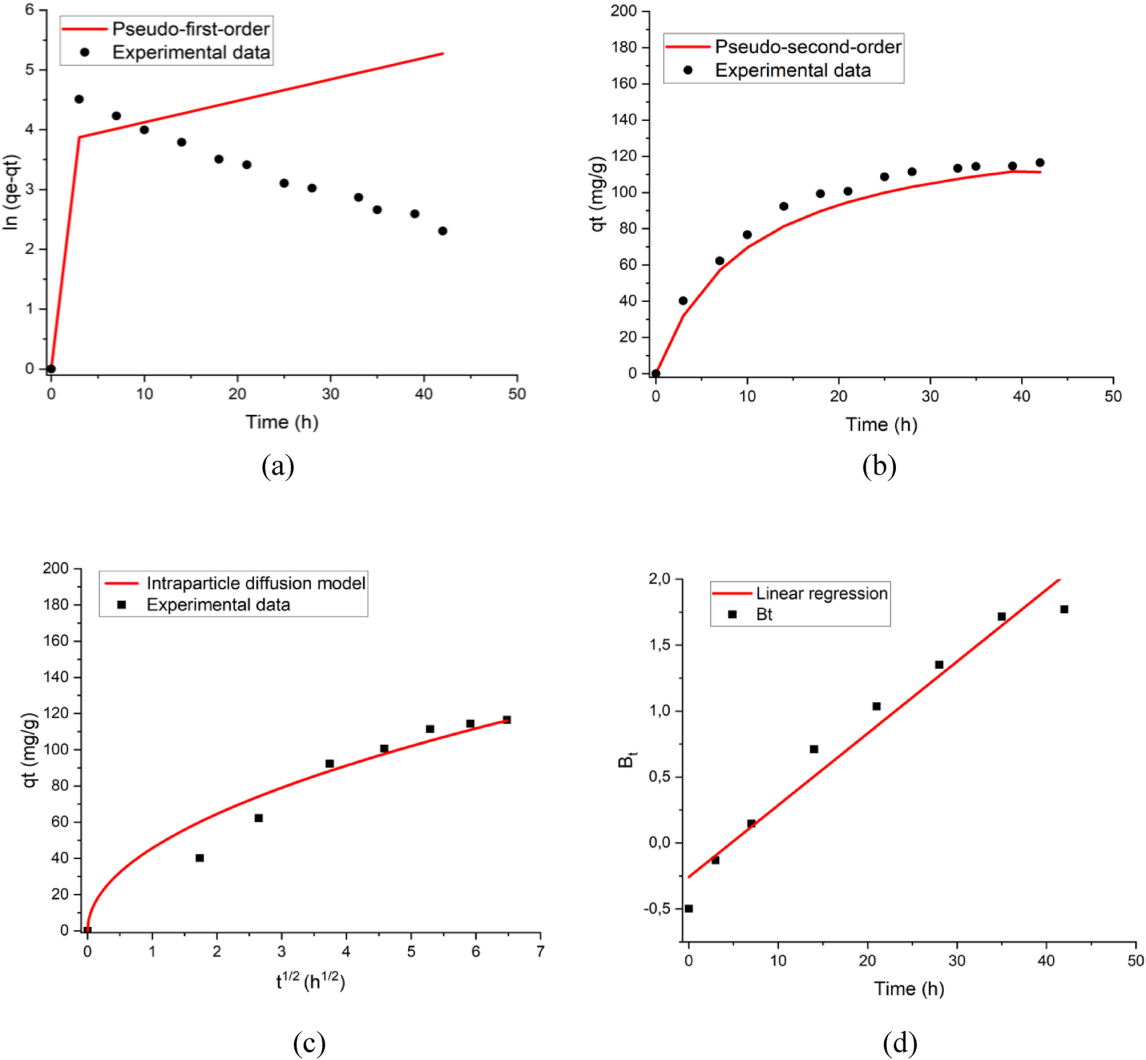
PFOA adsorption kinetics of PA-activated RHs modified with DBD plasma (initial PFOA concentration = 50 mg/L, adsorption time = 0–42 h). (**a**) Pseudo-first-order model and experimental data, (**b**) pseudo-second-order model and experimental data, (**c**) intraparticle diffusion mode and experimental data, and (**d**) Boyd model.

The parameters obtained from the adsorption kinetic experiments were summarized in Table S3. The pseudo-first-order model represents the condition with a few active sites existing in the adsorbent, while the pseudo-second-order model is suitable for abundant active sites in the adsorbent^86^. The sorption rate constant acquired from the pseudo-second-order (k_2_) models was 0.00141 g mg^−1^ min^−1^. The adsorption equilibrium can be reached after 14 h. Based on Fig. 7b, the pseudo-second-order kinetic model exhibited an outstanding fitting to the experimental kinetic data in accordance with the high correlation coefficient (R^2^ = 0.984). In contrast, the pseudo-first-order kinetic model (Fig. 7a) failed to interpret the rate data (R^2^ = 0.522) as it did not fit well with the adsorption data. These results indicate that the chemisorption mechanism is the primary controlling step for PFOA removal with RHs, as the observed kinetic rate values correspond with this mechanism^1^.

As shown in Fig. 7c, the intraparticle diffusion model is not linear across the entire time span. This multilinearity means that the PFOA adsorption process was not only governed by intraparticle diffusion. Generally, there are three main stages involved in the adsorption mechanism of PFOA as illustrated in Fig. S9. The initial stage with a deviation during 7 h revealed that the external mass transfer was more effective in the adsorption process during this period, whereas the PFOA moved from the bulk solution towards the external surface of RHs. The second stage was governed by intra-particle diffusion as the primary rate-controlling step, which is indicated by the regression line passing through the origin after 14 h which proceeded slowly due to the low concentration in the inner part of the RHs (the first stage reduced the amount of PFOA yielded)^1,86,87^. The intraparticle diffusion rate constant (k_d_) was 45.636 mg/g h^1/2^ and the C constant was more than zero which confirms that intraparticle diffusion is not the only rate-limiting step^88^. This statement is also ensured by the plot of the Boyd model (Fig. 7d) where the straight line did not pass through the origin points. The third stage is adsorption on active sites as the rate-controlling step as illustrated by the pseudo-second-order model. However, the adsorption kinetic is mostly affected by the pore size and particle diameter of adsorbents^67^.

### Possible functionalization mechanism of PA-activated plasma-treated RHs

**Figure 8:**
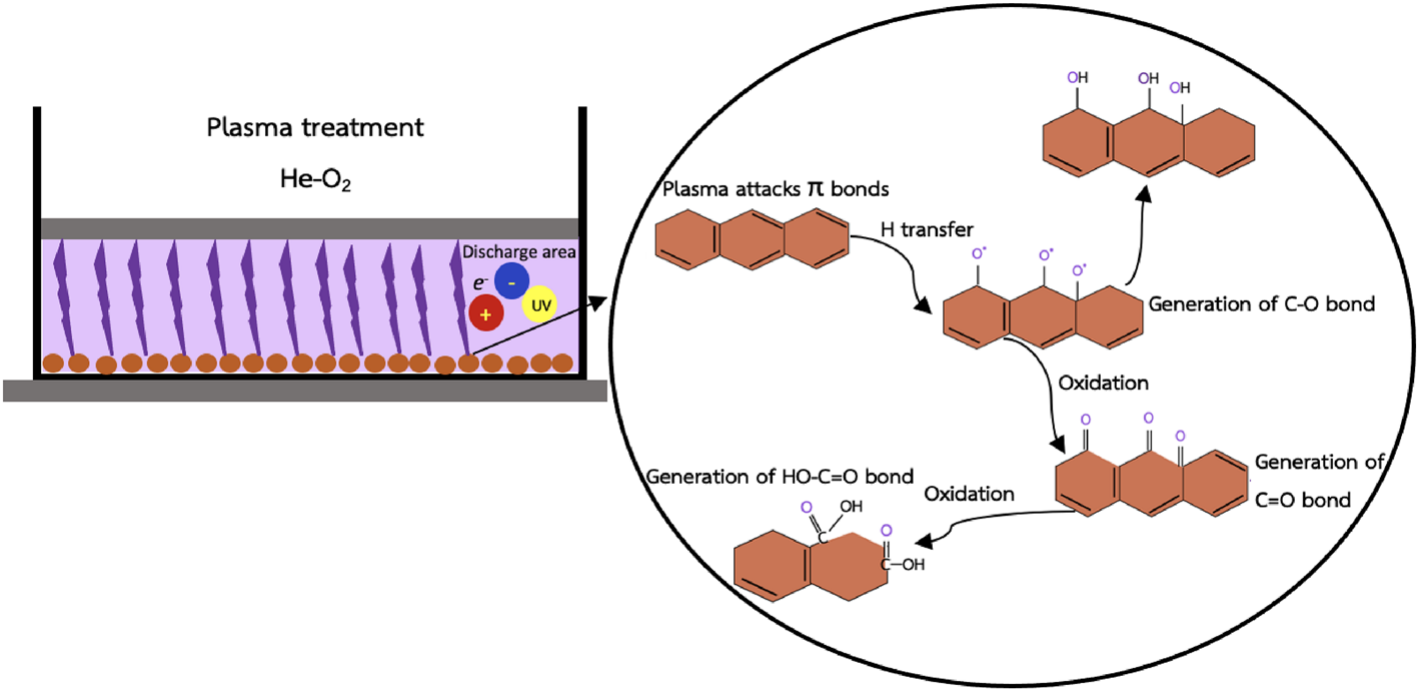
Proposed mechanism of RH functionalization with He–O_2_ plasma treatment.

The mechanism of plasma treatment is complex because many active species are involved. In general, the plasma surface modification mechanism consists of five processes: physical process due to ion bombardment, chemical process, plasma heating, high electrical strength, and UV-irradiation^16^. The most significant mechanism taking place during material modification is a combination of plasma-chemical and plasma-physical processes to modify the textural property and surface chemistry^14^. The application of the He-O_2_ gas mixture can improve the plasma treatment because electronegative O_2_ could produce strong oxidizing species, and the addition of He in the plasma oxidation process helps stabilize and ameliorate the uniformity of oxygen discharge^47^. The He–O_2_ gas is beneficial for plasma oxidation because, with the generation of ^•^OH, which is a vigorous and nonselective oxidizing agent, RHs can produce various chemical oxidation effects resulting in the generation of carboxyl, carbonyl, and hydroxyl groups^89^. During the plasma-physical process, the etching effect dominantly happened; meanwhile, the functional groups grafting effect prominently occurred in the plasma-chemical process. The proposed RH functionalization mechanism is visualized in Fig. 8. Firstly, the plasma bombarded the RH surface at the π bonds in C=C because these bonds are most vulnerable to plasma attack, leading to the generation of highly active sites (e.g. free radicals, metastable species, and positive ions) for binding functional groups^64,90,91^. At the beginning stage, further reaction with active oxygen atoms can generate C–O bonds, which are then stabilized by hydrogen atom transfer within the same or an adjacent chain and may produce C–OH bonds. The oxygen radicals were also created on the RH surface and can form new C=O (e.g. aldehyde) bonds via intramolecular rearrangement on the C–C bonds. C=O–OH can be formed because of the process involving the interaction between radical species produced by plasma treatment on the C=O bonds and active oxygen atoms, followed by stabilization through proton transfer^91^.

The mechanism described above is supported by the FTIR analysis as shown in Fig. 1. The spectrum exhibits a strong absorption peak at 1035 cm^–1^, indicating the presence of C−O bonds, and a peak at 1718 cm^–1^, suggesting the presence of C=O (–COOH) or carboxylic acid functional groups. Pristine RHs, which did not undergo plasma treatment, exhibited the lowest intensity of oxygenated functional groups compared to the plasma-treated RHs. Among the treated samples, the highest intensity of functional groups was observed in the PA + 30% O_2_ plasma sample. The XPS results depicted in Fig. S3 and Table 1 further support these findings. After plasma treatment, the concentration of oxygen gas increased, and the amount of C=O and COOH groups continues to increase, indicating the successful generation of oxygenated functional groups on the RH surface as a result of plasma treatment.

Then, the functional groups that were just generated spread out to some spaces in RH pores. The variation of functional groups depends upon certain parameters such as plasma discharge power, type of carrier gas, treatment time, and pressure^92^. The reaction between highly active species and RH surface can form volatile substances, enlarge micropores, and possibly break covalent C=C bonds, so the increase in plasma modification time intensifies the destructive etching effect^64^. Due to the violent etching effect, the incoming ions can penetrate the RH surface, increasing the surface roughness significantly. The loading of –OH and –COOH functional groups on RHs can improve the hydrogen content, which is beneficial for hydrogen bond reactions with PFOA^89^. Nevertheless, these functional groups have the potential to be destroyed by highly energetic ions^90^.

## Conclusions

The utilization of He–O_2_ DBD plasma treatment can significantly improve the adsorbability of RHs to adsorb PFOA in water. The optimal DBD plasma treatment condition was He + 30 vol.% O_2_ gas mixture, 1.5 L/min total flow rate, and 10 min treatment time at 100 W of discharge power. The maximum PFOA removal efficiency was found to be 96.69% with a maximum uptake capacity of 565 mg/L when the Langmuir isotherm model was applied. The lower removal efficiency was observed at the final pH > 4, indicating that electrostatic repulsion dominantly happened between the treated RHs and PFOA. Varying the solution pH, the surface charge on the adsorbent can be altered through the protonation and deprotonation of oxygen functional groups. The SEM observation revealed various pore generations on PA-activated plasma-treated RH surfaces resulting in higher surface area than untreated RHs, but overexposure of plasma for more than 15 min led to pore wall destruction and reduction in surface area. The FTIR investigation revealed that the plasma treatment can produce more oxygen-containing functional groups (hydroxyl, carboxyl, etc.) than pristine RHs, which was favorable for PFOA adsorption. The adsorption equilibrium isotherms fitted the Langmuir model and the adsorption kinetic fitted the pseudo-second-order model. The findings of this research offer a high removal efficiency biosorbent to remove PFOA from water. Consequently, it is likely that this novel treatment technique would also become an important tool for modifying other adsorbents and applying on other pollutants.

### Supplementary Information

Supplementary Information.

## Data Availability

The datasets used and/or analyzed during the current study are available from the corresponding author upon reasonable request.
