# Supplementary material for: Highly effective removal of perfluorooctanoic acid (PFOA) in water with DBD-plasma-enhanced rice husks
Source: Sci Rep. 2023 Aug 14;13:13210. doi: 10.1038/s41598-023-40197-3 (PMC10425357; doi:10.1038/s41598-023-40197-3)
Supplement: Supplementary file 1 — Supplementary Information. [file 41598_2023_40197_MOESM1_ESM.docx]

Supporting Information

**Highly Effective Removal of Perfluorooctanoic Acid (PFOA) in Water with DBD-Plasma-Enhanced Rice Husk**

Thera Sahara^a^, Doonyapong Wongsawaeng^a,*^, Kanokwan Ngaosuwan^b^, Worapon Kiatkittipong^c^, Peter Hosemann^d^, Suttichai Assabumrungrat^e,f^

*^a^Research Unit on Plasma Technology for High-Performance Materials Development, Department of Nuclear Engineering, Faculty of Engineering, Chulalongkorn University, Bangkok 10330, Thailand*

*^b^Division of Chemical Engineering, Faculty of Engineering, Rajamangala University of Technology Krungthep, Bangkok 10120, Thailand*

*^c^Department of Chemical Engineering, Faculty of Engineering and Industrial Technology, Silpakorn University, Nakhon Pathom 73000, Thailand*

*^d^Department of Nuclear Engineering, Faculty of Engineering, University of California at Berkeley, 94720, U.S.A.*

*^e^Center of Excellence in Catalysis and Catalytic Reaction Engineering, Department of Chemical Engineering, Faculty of Engineering, Chulalongkorn University, Bangkok 10330, Thailand*

*^f^Bio-Circular-Green-economy Technology & Engineering Center (BCGeTEC), Faculty of Engineering, Chulalongkorn University, Bangkok, Thailand 10330*

| Compound | Molecular weight (g/mol) | Water solubility (mg/L) [25] | CMC (mg/L) [48] | pKa [47] |
| --- | --- | --- | --- | --- |
| PFOA | 414.07 | 3,400 | 15,696 | 2.5 |

**Table S1** Physical properties of PFOA

**Table S2** Parameters of adsorption isotherm study

| **Langmuir model** | | | | **Freundlich model** | | |
| --- | --- | --- | --- | --- | --- | --- |
| q_m_ (mg/g) | K_l_ (L/mg) | R_L_ | R^2^ | K_f_ | 1/n | R^2^ |
| 565 | 0.056 | 0.261 | 0.972 | 33.611 | 0.534 | 0.875 |

**Table S3** Parameters of adsorption kinetic study

| **Pseudo-first-order model** | | | **Pseudo-second-order model** | | | **Intraparticle diffusion model** | | | **Boyd model** | |
| --- | --- | --- | --- | --- | --- | --- | --- | --- | --- | --- |
| q_e_ (mg/g) | k_1_ | R^2^ | q_e_ (mg/g) | k_2_ | R^2^ | k_d_ (mg/g h^1/2^) | C (mg/g) | R^2^ | Slope | R^2^ |
| 12.109 | 0.00188 | 0.522 | 131.579 | 0.00141 | 0.984 | 45.636 | 4.48 $\times$10^-13^ | 0.949 | 0.054 | 0.960 |


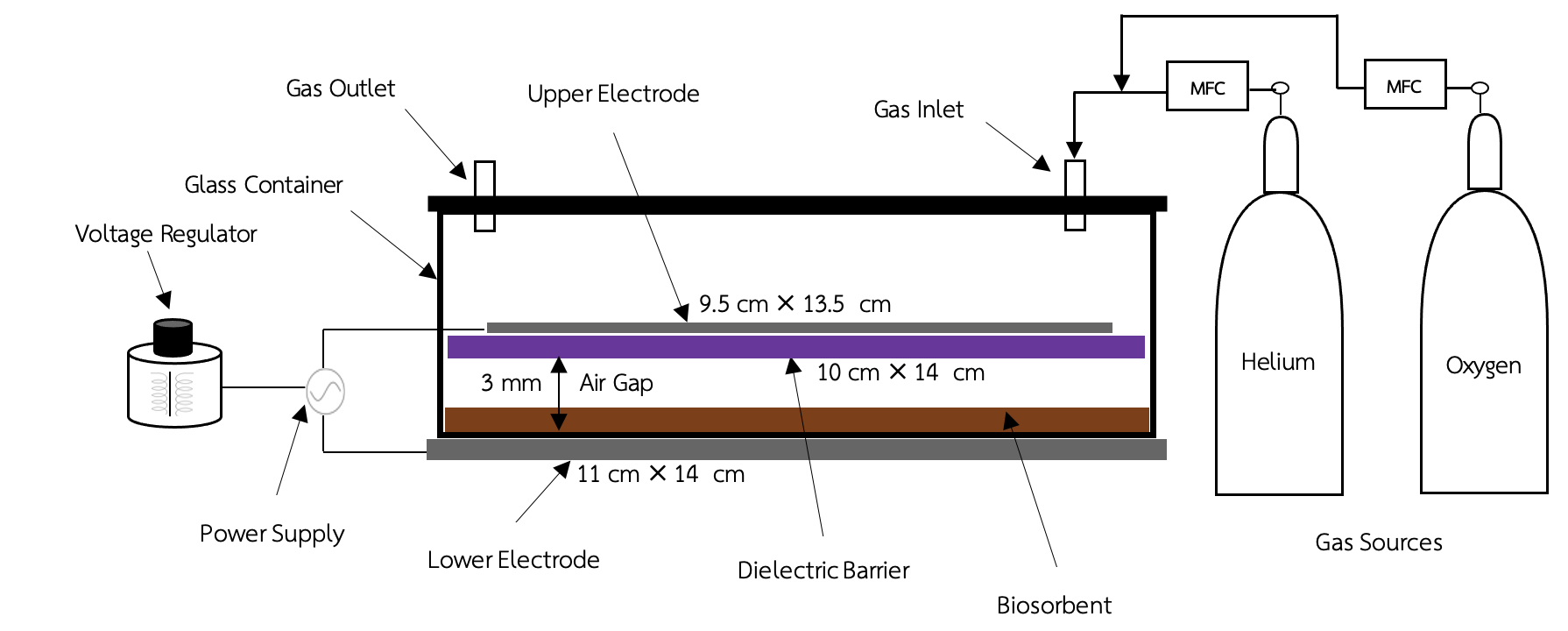


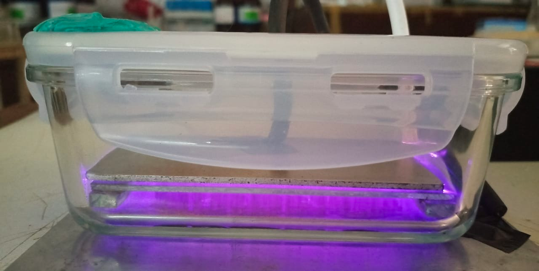
(a)

(b)

**Fig. S1** (a) Illustration of experimental setup used for DBD plasma treatment and (b) plasma glow discharge inside DBD plasma reactor.


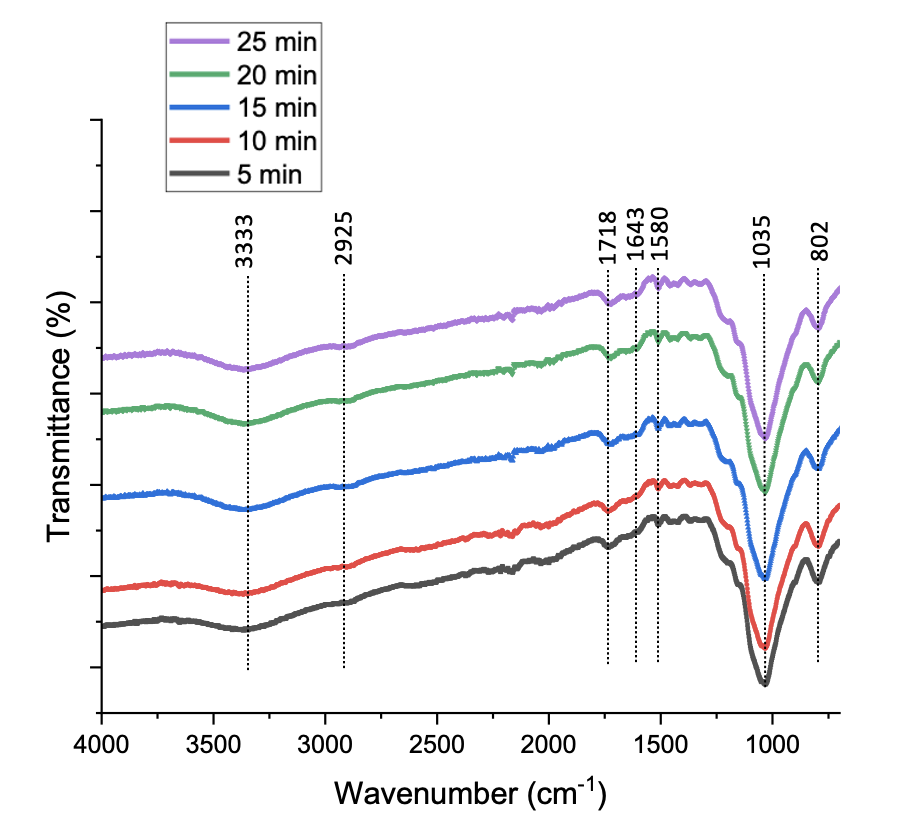


**Fig. S2** FTIR spectra of PA + 30% O_2_ plasma-treated RHs with variations of plasma treatment time at 100 W and 1.5 L/min total gas flow rate.


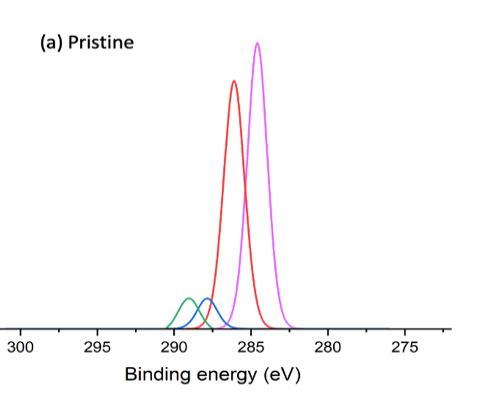

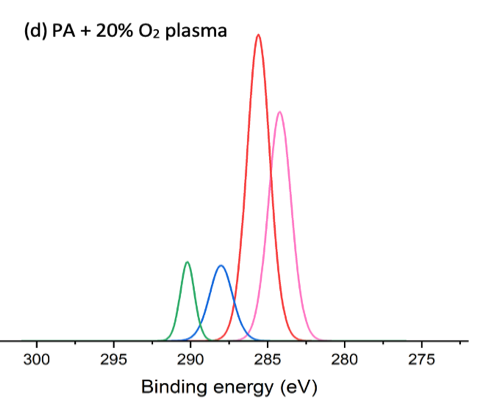

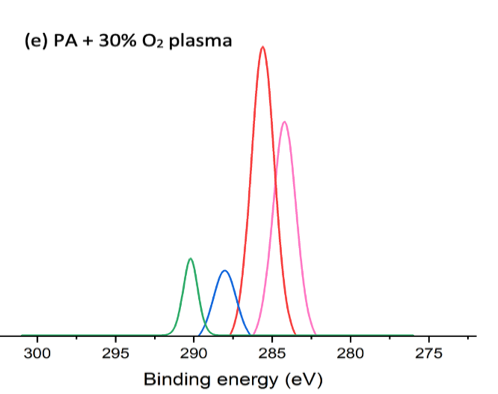

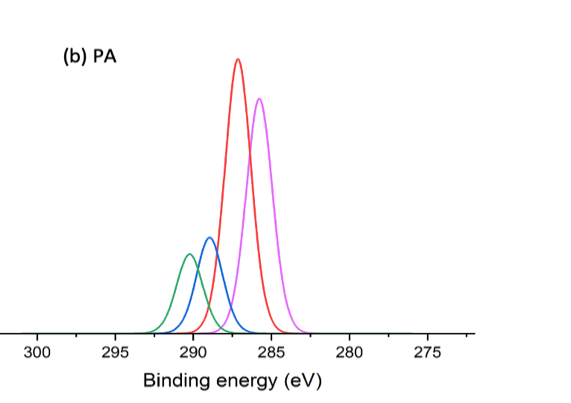

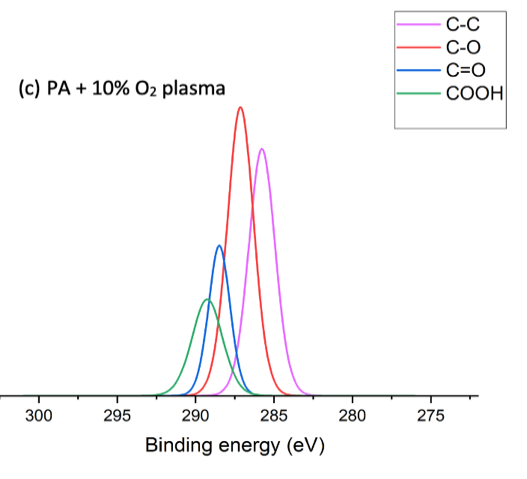

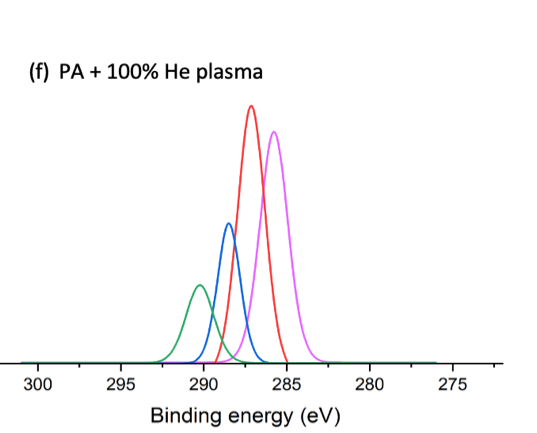


**Fig. S3** XPS C1s spectra of (a) Pristine RHs, (b) PA, (c) PA + 10% O_2_ plasma, (d) PA + 20% O_2_ plasma, (e) PA + 30% O_2_ plasma, and (f) PA + 100% He plasma (10 min plasma treatment time, 100 W, and 1.5 L/min total gas flow rate).


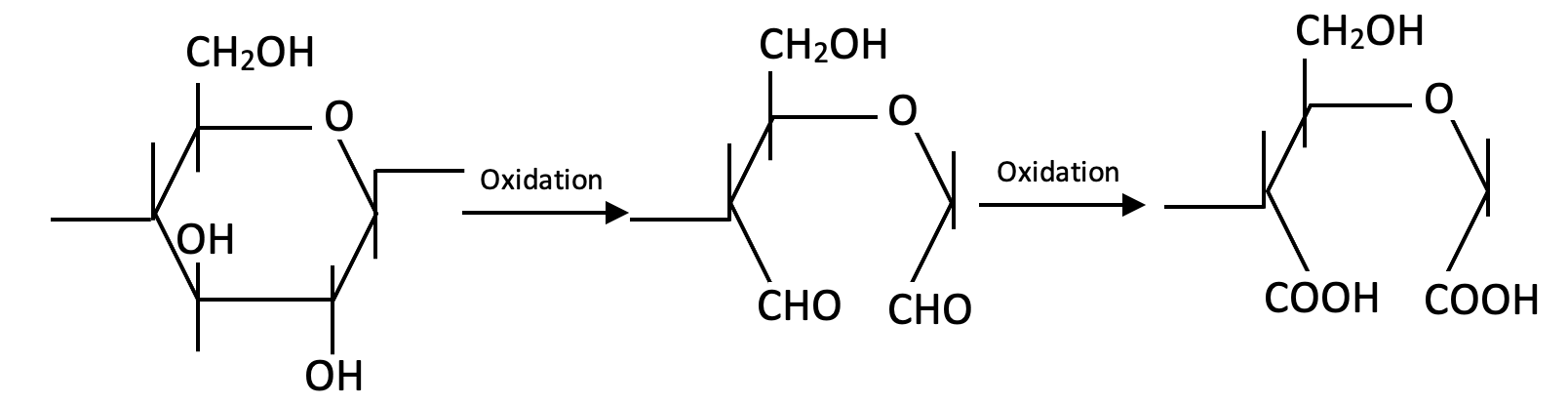


**Fig. S4** Proposed oxidation reaction of cellulose on PA-activated plasma-treated RH surface.


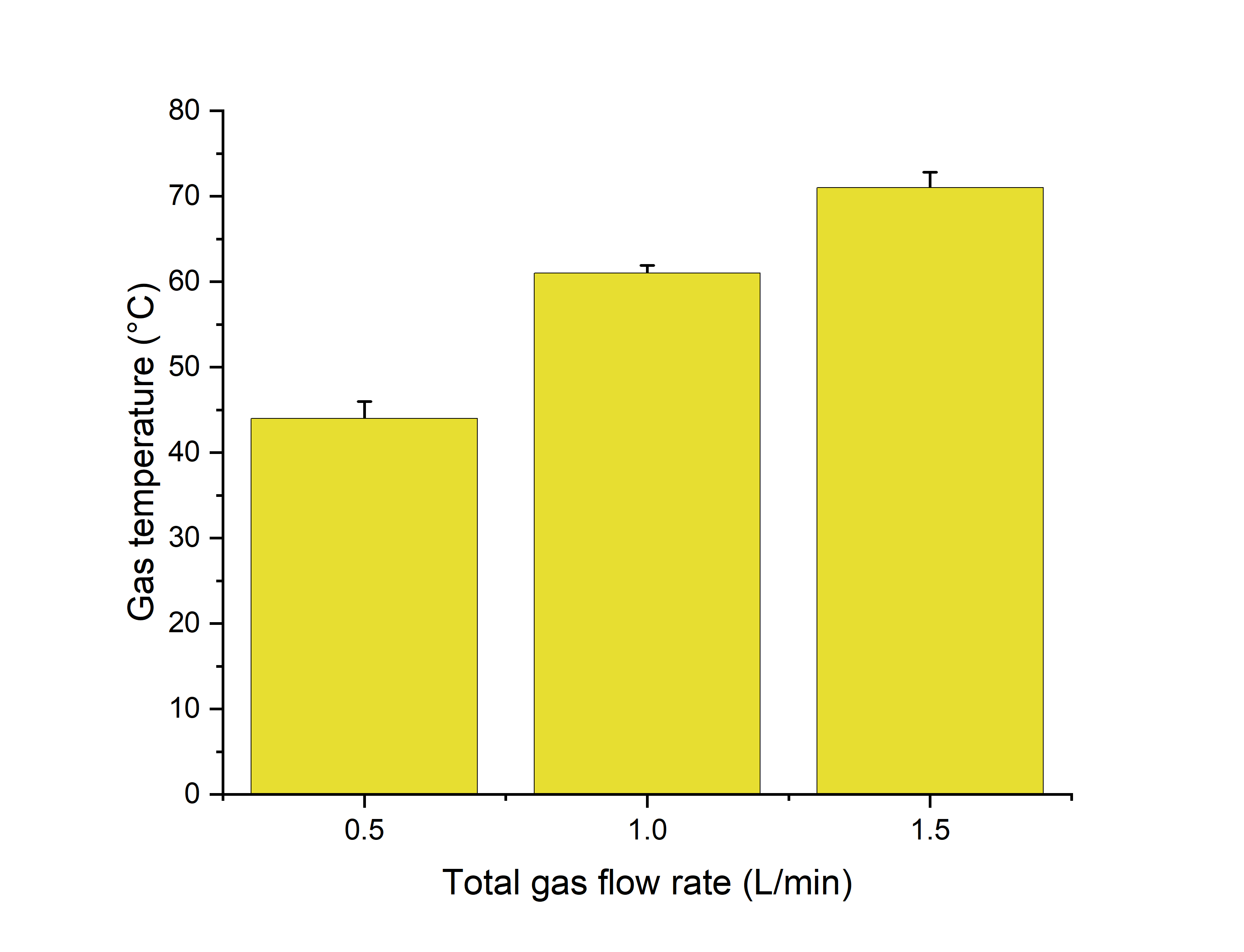


**Fig. S5** Effects of total gas flow rate on gas temperature (10 min treatment time at 100 W).


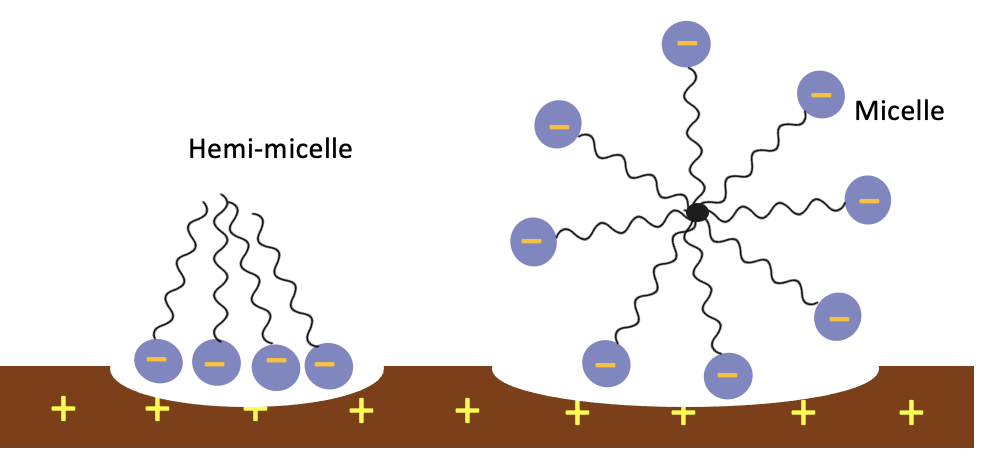


**Fig. S6** Drawing of micelle and hemi-micelle aggregate establishment on plasma-treated RH.


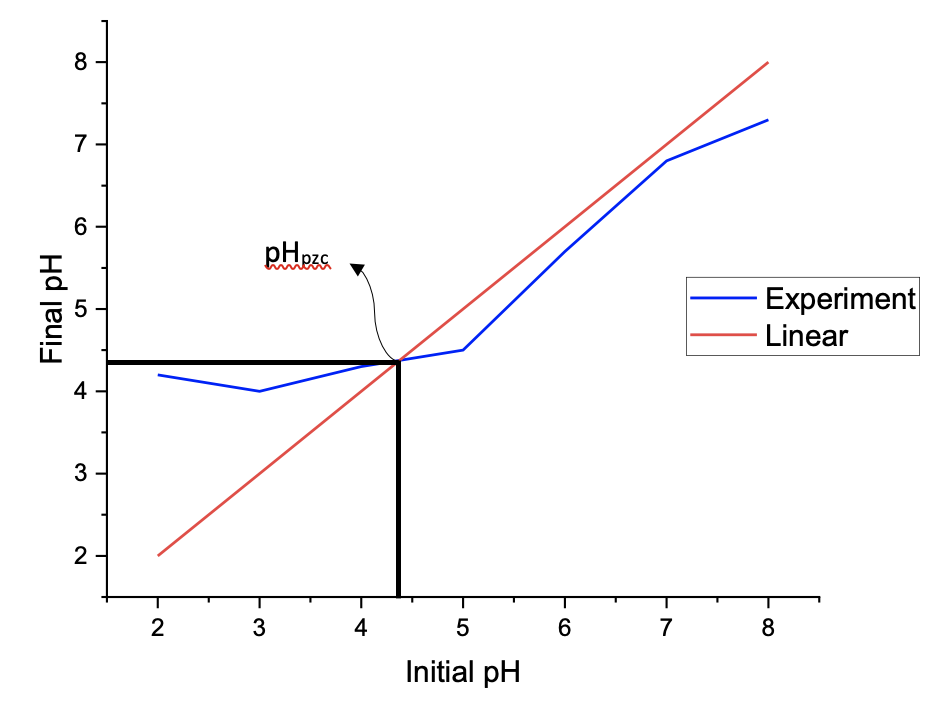


(a)


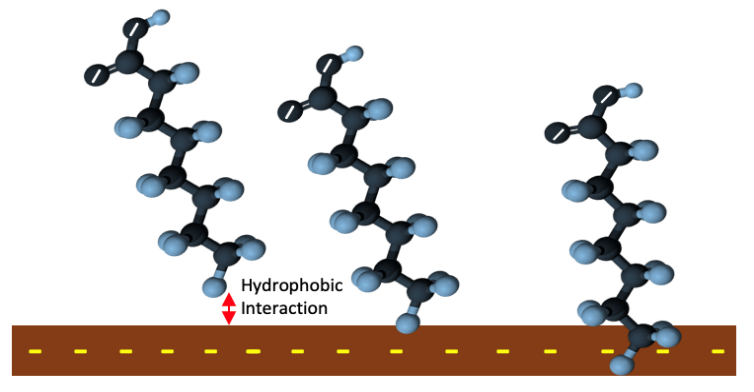


(b)

(c)


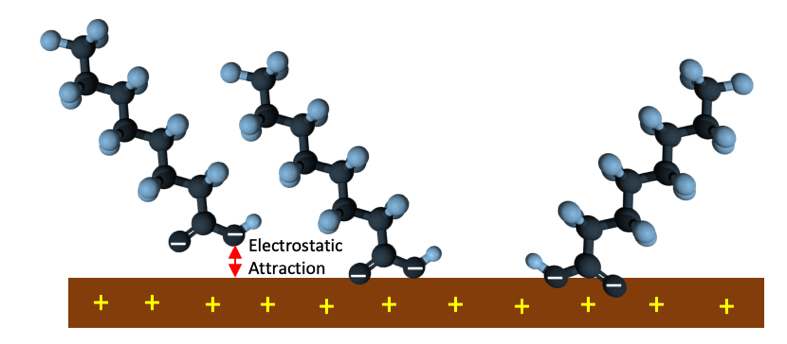


**Fig. S7** (a) pH_pzc_ measurement with pH drift method (He + 30 vol.% O_2_, 1.5 L/min total flow rate, 10 min, and 100 W), (b) drawing of electrostatic attraction in PFOA adsorption, and (c) drawing of hydrophobic interaction in PFOA adsorption.


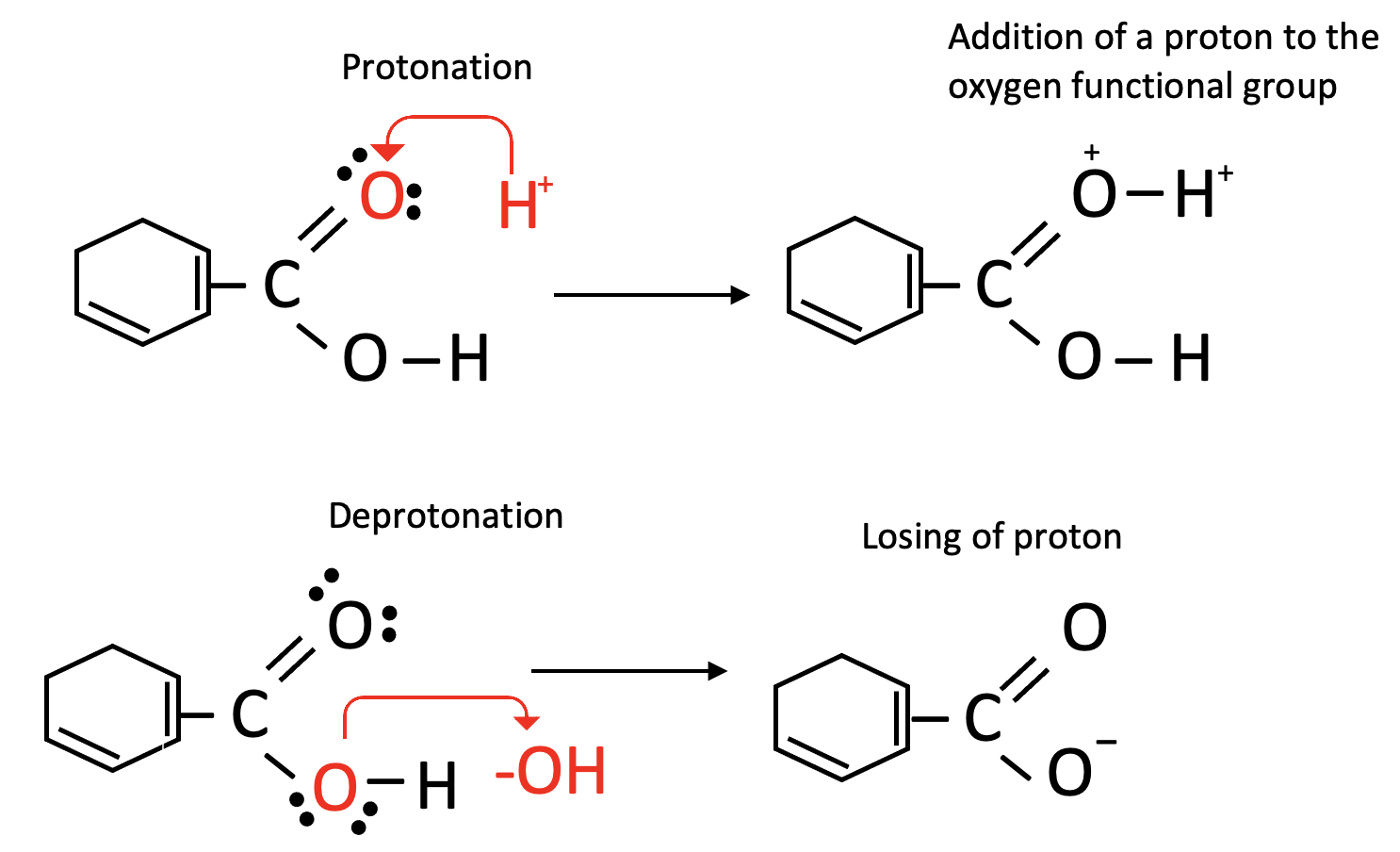


**Fig. S8** Illustration of protonated and deprotonated carboxylic acid of RHs.


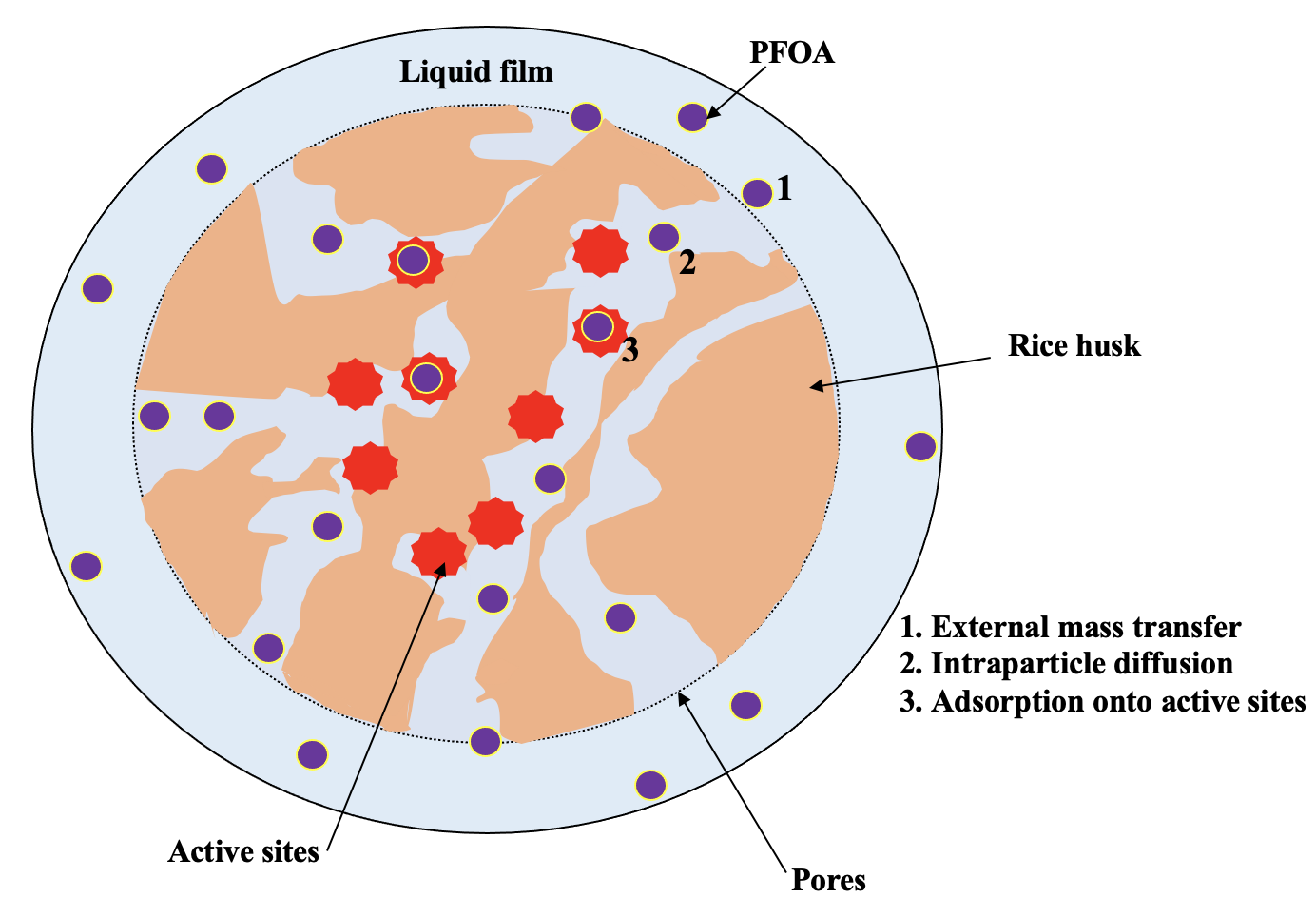


**Fig. S9** Illustration of adsorption mass transfer steps
